# Differences in mood instability in patients with bipolar disorder type I and II: a smartphone-based study

**DOI:** 10.1186/s40345-019-0141-4

**Published:** 2019-02-01

**Authors:** Maria Faurholt-Jepsen, Mads Frost, Jonas Busk, Ellen Margrethe Christensen, Jakob E. Bardram, Maj Vinberg, Lars Vedel Kessing

**Affiliations:** 1 Copenhagen Affective Disorder Research Center (CADIC), Psychiatric Center Copenhagen, Rigshospitalet, Blegdamsvej 9, 2100 Copenhagen, Denmark; 2 IT University of Copenhagen, Rued Langgaards Vej 7, 2300 Copenhagen, Denmark; 3 Department of Applied Mathematics and Computer Science, Technical University of Denmark, Kongens Lyngby, Denmark

**Keywords:** Bipolar disorder, Hypomania, Depression

## Abstract

**Background:**

Mood instability in bipolar disorder is associated with a risk of relapse. This study investigated differences in mood instability between patients with bipolar disorder type I and type II, which previously has been sparingly investigated.

**Methods:**

Patients with bipolar disorder type I (n = 53) and type II (n = 31) used a daily smartphone-based self-monitoring system for 9 months. Data in the present reflect 15.975 observations of daily collected smartphone-based data on patient-evaluated mood.

**Results:**

In models adjusted for age, gender, illness duration and psychopharmacological treatment, patients with bipolar disorder type II experienced more mood instability during depression compared with patients with bipolar disorder type I (*B: 0.27, 95% CI 0.007; 0.53, p *= *0.044*), but lower intensity of manic symptoms. Patients with bipolar disorder type II did not experience lower mean mood or higher intensity of depressive symptoms compared with patients with bipolar disorder type I.

**Conclusions:**

Compared to bipolar disorder type I, patients with bipolar disorder type II had higher mood instability for depression. Clinically it is of importance to identify these inter-episodic symptoms. Future studies investigating the effect of treatment on mood instability measures are warranted.

*Trial registration* NCT02221336

## Background

Bipolar disorder is characterized by recurrent episodes of depression and (hypo)mania and in the form of sub-syndromal mood swings. A substantial proportion of patients with bipolar disorder does not experience full remission, remains symptomatic during inter-episode periods and experience daily mood swings above what is experienced by healthy individuals (Bopp et al. 2010; Judd et al. 2003; Joffe et al. 2004). Mood instability between affective episodes is associated with substantial disability and poor prognostic factors such as increased risk of hospitalization, high risk of relapse, and impaired functioning (Bopp et al. 2010; Judd et al. 2003; Joffe et al. 2004; Kupka et al. 2007; MacQueen et al. 2003; Strejilevich et al. 2013; Patel et al. 2015). This emphasizes the serious nature of bipolar disorder. Noticing the clinically and functionally impact of mood instability, mood instability has been suggested as a target for treatment and may be a more sensitive outcome measure in randomized controlled maintenance trials (RCT) than for example relapse or recurrence of affective episodes (Bopp et al. 2010; Bonsall et al. 2012; Saunders et al. 2016). However, few studies have suggested methods and measures on how to investigate mood instability and its possible predictors.

Patients with bipolar disorder type II seem to spend more time depressed, less time euthymic and experience greater depression instability than patients with bipolar disorder type I (Joffe et al. 2004; Faurholt-Jepsen et al. 2015a; O’Donnell et al. 2018; Vinberg et al. 2017). Previous studies investigating differences in mood between bipolar disorder type I and II have collected data on mood on a weekly and not daily basis and furthermore used paper based charts, increasing the risk of recall bias and low compliance (Judd et al. 2003; Joffe et al. 2004; Kupka et al. 2007; MacQueen et al. 2003; Strejilevich et al. 2013). One previous pilot study by the authors used smartphone-based daily electronic self-monitoring of mood (Faurholt-Jepsen et al. 2015a). In this study (the MONARCA I trial) including 33 patients with bipolar disorder, the patients with bipolar disorder type II spend less time euthymic, a higher proportion of time with depressive symptoms and experienced higher mood instability for depression compared with patients with bipolar disorder type I (Faurholt-Jepsen et al. 2015a). Apart from this previous pilot study by the authors, no study has investigated differences in mood instability between patients with bipolar disorder type I and bipolar disorder type II using fine-grained daily electronic data. A recent paper discussed differences in momentary and retrospective trait self-report techniques pointing out that retrospective self-monitoring is influenced by peak moments with greater salience of moments that occur closest in time to the assessment (Conner and Barrett 2012), which may be a particular problem when investigating mood instability given its dynamic structure in both polarity, variation and intensity. Ecological momentary assessments (EMA) reflect the methods used to collect assessments of individual’s real-time states repeatedly over time and during naturalistic settings, and may reduce recall bias (Shiffman et al. 2008). Smartphones extends the use of EMA beyond its classical use for self-reports and offer the opportunity to collect fine-grained data unobtrusively and outside the clinical settings (Ebner-Priemer and Trull 2009), and enable collection of data on daily subsyndromal mood fluctuations. Based on daily smartphone-based self-monitoring data, the present report aimed to investigate, and replicate findings from the before mentioned study by the authors (Faurholt-Jepsen et al. 2015a). We hypothesized that patients with bipolar disorder type II had lower mean mood level, spend less time euthymic, a higher proportion of time with depressive symptoms, experienced higher instability for depression, i.e. more fluctuations in self-monitored depressive mood, and experienced higher levels and intensity of depressive symptoms compared with patients with bipolar disorder type I.

## Methods

### Design, settings and participants

The patients were recruited as part of a randomized controlled single-blind parallel-group trial investigating the effect of smartphone-based self-monitoring, the MONARCA II trial (Faurholt-Jepsen et al. 2014) including adult patients with bipolar disorder for a 9 months follow-up period. All patients with a diagnosis of bipolar disorder who previously had been treated at The Copenhagen Clinic for Affective Disorders, Denmark in the period 2004 to January 2016, currently treated at community psychiatric centers, private psychiatrists and general practitioners were invited to participate in the trial, corresponding to approximately 735 potential participants. Treatment at the Copenhagen Clinic for Affective Disorders comprised combined psychopharmacological treatment as according to international guidelines, group based psychoeducation and supporting therapy for a total of 2-year (Kessing et al. 2013).

Inclusion criteria: Bipolar disorder diagnosis according to ICD-10 using the schedules for clinical assessments in neuropsychiatry (SCAN) (Wing et al. 1990) and previously treatment at the Copenhagen Clinic for Affective Disorders. Exclusion criteria: schizophrenia, schizotypal or delusional disorders; previous use of the smartphone-based monitoring system; pregnancy and lack of Danish language skills.

Patients were diagnostically categorized into bipolar disorder type I or II according to the SCAN interview. During the study the patients’ severity of depressive and manic were evaluated at baseline, after 4 weeks, 3 months, 6 months and 9 months using the Hamilton Depression Rating Scale 17-items (HDRS) (Hamilton 1967) and the Young Mania Rating Scale (YMRS) (Young et al. 1978).

### Smartphone-based monitoring

Patients were randomized to using a smartphone-based monitoring system (the MONARCA II system) for daily self-monitoring (intervention group) or treatment as usual (control group). Only patients included in the intervention group collecting daily smartphone-based self-monitoring data were included and presented in the present report.

Patients in the intervention group used a smartphone with the MONARCA II app installed and were instructed to use the system for daily evaluation for 9 months. The MONARCA II system allowed for evaluation of the following measures on a daily basis: mood (scored on a scale from − 3 to + 3); sleep duration (measured in half-hour intervals); medicine intake; activity level (scored on a scale from − 3 to + 3); mixed mood (scored on a scale from 1 to 3); irritability (scored on a scale from 1 to 3); anxiety (scored on a scale from 1 to 3); cognitive problems (scored on a scale from 1 to 3); alcohol consumption; and stress (scored on a scale from 1 to 3). The patients were prompted to evaluate by an alarm set at a self-chosen time during the day. Further details regarding the MONARCA II system are described elsewhere (Faurholt-Jepsen et al. 2014).

### Statistical methods

The hypotheses and statistical analyses were defined á priori. The validity of self-monitored mood compared with clinically rated depressive and manic reflected by the HDRS and the YMRS, respectively was investigated. Since the HDRS and the YMRS reflects clinically rated severity of depressive and manic symptoms for the past 4 days, the mean self-monitored mood for the days the scales were reflecting were used in the analyses of the validity of self-monitored mood. A two-level linear mixed effect model, which accommodates both variation of the variables of interest within patients (intra-individual variation) and between individuals (inter-individuals variation) was employed. The models including a fixed effect of visit number (baseline, 4 weeks, 3 months, 6 months, 9 months) and a patients-specific random effect allowing for individual intercept and a slope for each participant were employed. Level one represented repeated measures of clinically rated symptoms (HDRS and YMRS) and level two represented inter-individual variation.

Differences in mean sleep duration, mood level and proportion of time with mixed symptoms were investigated by means of analysis of covariance (ANCOVA). To summarize the patient profiles over time summary measures or indexes covering three areas (instability, symptom load and intensity) as indicators of illness activity were calculated for each patient in the same way as in our previous paper based on daily self-monitored data on mood scores (Faurholt-Jepsen et al. 2015a) and as suggested and used by others (Strejilevich et al. 2013) (data presented in Table 2):

1. Mood instability factors: A mood instability factor measure was calculated as the number of mood changes divided by the number of weeks followed. A mood instability factor for depression was calculated as the number of mood changes of depressive polarity divided by the number of weeks followed. A mood instability factor for mania was calculated as the number of mood changes of manic polarity divided be the number of weeks followed.
2. Mood symptomatic factors: A mood symptomatic factor for depression was calculated as the number of days with depressive symptoms < − 0.5 on a mood scale from − 3 to + 3 divided by the number of weeks followed. A mood symptomatic factor for mania was calculated as the number of days with manic symptoms > + 0.5 on a mood scale from − 3 to + 3 divided by the number of weeks followed.
3. Mood intensity: A mood intensity factor for depression was calculated as the summary score of depressive symptoms divided by the number of weeks followed. A mood intensity factor for mania was calculated as the summary score of manic symptoms divided by the number of weeks followed.

In addition to these measures and as suggested by others, mood instability scores reflecting the extent to which consecutively measured mood differ from one another during the follow-up period were computed for each user by applying the root mean square successive difference (rMSSD) method, taking the square root of the sum of the squared differences between daily and previous day mood scores (O’Donnell et al. 2018; Ebner-Priemer and Trull 2009). The rMSSD reflects both size and temporal order of changes in mood. Higher rMSSD scores indicate more instability. Previous reports have demonstrated the construct validity of this method (Ebner-Priemer and Trull 2009; Jahng et al. 2008).

Regression analyses were used to evaluate the relationship between the different calculated mood indexes and bipolar disorder type I or bipolar disorder type II, respectively. First, we considered an unadjusted model (model 1). Secondly, we considered a model adjusted for age and gender (model 2). Thirdly, we considered a model adjusted for age, gender, illness duration and psychopharmacological treatment (anticonvulsant treatment yes/no, antipsychotic treatment yes/no, antidepressant treatment yes/no, lithium treatment yes/no) (model 3) as these variables could affect the differences in mood instability measures between the two groups. Model assumptions were checked for each of the statistical analyses. As few prior studies have investigated differences in mood instability indexes between bipolar disorder type I and II we were not able to make statistical power analyses prior to the study since potential effects were unknown. The overall hypotheses were made a priori based on our prior findings within patients with bipolar disorder. Nevertheless, as these hypotheses included several measures on mood instability, the present study should be considered as hypotheses generating and the results needs further replication. Consequently, we did not account for multiple testing in the statistical models. p-values below 0.05 were considered statistical significant. Data were entered using Excel and Epidata^®^, STATA (StataCorp LP, Collega Station, TX, USA) version 12.1 was used for statistical analyses.

### Ethical considerations

Ethical permission was obtained from the Regional Ethics Committee in The Capital Region of Denmark and The Danish Data Protection Agency (H-2-2014-059).

## Results

### Background characteristics

From October 2014 to January 2018, a total of 85 patients collected daily smartphone-based self-monitoring data (the intervention group of the MONARCA II trial) for a study period of 9 months. In total 84 patient provided self-monitoring data during the study and were included in the present study (bipolar disorder type I n = 53, bipolar disorder type II n = 31). As presented in Table 1, patients had a mean age of 43.0 years (SD 12.3) and 51 (61.2%) were of female gender. Patients with bipolar disorder type I were more often in prescribed treatment with antipsychotics compared with patients with bipolar disorder type II (bipolar disorder type I: 59.3%; bipolar disorder type II: 32.3%) (Table 1).

**Table 1:** Background characteristics of patients with bipolar disorder I and II, N = 84

	All patients (*n *= 84)	Bipolar disorder type I (*n *= 53)	Bipolar disorder type II (*n *= 31)	p
Age (years)	43.0 (12.3)	42.8 (11.9)	43.4 (13.3)	0.85
Female gender, % (n)	61.2 (51)	64.8 (34)	54.8 (17)	0.36
Full time employment, % (n)	17.7 (15)	16.7 (9)	19.4 (6)	0.73
Illness duration (years)	18.0 (10.1)	17.7 (10.6)	18.6 (9.5)	0.71
Number of previous depressive episodes	4 [2–10]	3.5 [2–9]	5 [3–10]	0.21
Number of previous (hypo)manic episodes	3 [2–7]	3 [2–6]	5 [2–8]	0.33
Hamilton depression rating scale score, baseline	7.6 (5.0)	7.9 (5.0)	7.2 (6.0)	0.57
Young Mania Rating Scale score, baseline	3.2 (4.6)	4.1 (5.6)	2.6 (3.0)	0.16
Psychopharmacological treatment				
Anticonvulsants, % (n)	55.9 (47)	57.4 (30)	54.8 (17)	0.82
Antipsychotics, % (n)	48.8 (41)	59.3 (31)	32.3 (10)	0.017
Antidepressants, % (n)	22.6 (19)	26.4 (14)	16.1 (5)	0.28
Lithium, % (n)	63.1 (53)	68.5 (36)	54.8 (17)	0.21

Data are mean (SD), median [IQR] or proportions (n) unless otherwise stated

At baseline a total of 52.9% (n = 44) of the patients had a HDRS score ≤ 7 and 87.1% (n = 73) had a HDRS score ≤ 14. A total of 85.3% (n = 72) had a YMRS score ≤ 7 and 97.6% (n = 82) had a YMRS score ≤ 14. During the entire 9 months follow-up period 85.6% (n = 72) of the patients had a HDRS score ≤ 14 and 97.5% (n = 82) had a YMRS score ≤ 14. During the study patients adhered to self-monitoring with a mean of 72.6% (a mean of 190 days) of the days. Data in the present report are based on 15.975 daily smartphone-based self-monitored observations.

Regarding the validity of self-monitored mood compared with clinically rated depressive and manic symptoms, there was a statistically significant negative association between self-monitored mood and scores on the HDRS in both unadjusted models and in models adjusted for age, gender and psychopharmacological treatment (adjusted model B: − 0.033, 95% CI − 0.046; − 0.20, p < 0.0001). Thus, for every increase of 10 points on the HDRS there was a decrease of 0.33 point on the self-monitoring mood scale (range − 3 to + 3). Also, there was a statistically significant positive association between self-monitored mood and scores on the YMRS in both unadjusted models and in models adjusted for age, gender and psychopharmacological treatment (adjusted model B: 0.044, 95% CI 0.023; 0.066, p < 0.0001). Thus, for every increase of 10 points on the YMRS there was an increase of 0.44 point on the self-monitoring mood scale (range − 3 to + 3). Analyses including BD type (BD type I or II) did not alter the estimates.

### Bipolar disorder subtype and self-monitored smartphone data

Overall, regardless the bipolar subtype, the patients had a mean mood level below zero and reported mixed symptoms a large proportion of time when experiencing levels of depression or mania during the study, but there were no statistically significant differences (all p-values > 0.05) between patients with bipolar disorder type I and bipolar disorder type II in the self-monitored mean mood level [patients with bipolar disorder type I: − 0.15 (95% CI − 0.26; − 0.043) vs. patients with bipolar disorder type II: − 0.20 (95% CI − 0.33; − 0.065)], the proportion of overall time with mixed symptoms [patients with bipolar disorder type I: 8.77% (95% CI 4.75; 15.63) vs. patients with bipolar disorder type II: 3.51% (95% CI 0.86; 13.23)], the proportion of time with mixed symptoms when scoring < − 0.5 on a mood scale [patients with bipolar disorder type I: 16.67% (95% CI 8.07; 41.40) vs. patients with bipolar disorder type II: 11.11% (95% CI 1.45; 37.73)] and the proportion of time with mixed symptoms when scoring > + 0.5 om a mood scale [patients with bipolar disorder type I: 25.00% (95% CI 4.56; 54.56) vs. patients with bipolar disorder type II: 16.66% (95% CI 5.95; 26.18)].

Patients with bipolar disorder type II had a statistically significantly lower mean sleep duration per night (hours) compared with patients with bipolar disorder type I (6.54 (95% CI 6.18; 6.90) vs. 7.27 (95% CI 6.98; 7.57) vs., p = 0.036).

### Bipolar disorder subtype and mood instability

Unadjusted and adjusted regression models on indexes reflecting mood instability measures are presented in Table 2. As hypothesized, patients with bipolar disorder type II had statically significantly higher mood instability factor for depression (models adjusted for age, gender, illness duration and psychopharmacological treatment (anticonvulsant treatment yes/no, antipsychotic treatment yes/no, antidepressant treatment yes/no, lithium treatment yes/no): *B: 0.11, 95% CI 0.0085; 0.21, p *= *0.034*) compared with patient with bipolar disorder type I.

**Table 2:** Differences in mood instability indexes reflecting self-monitored illness activity in patients with bipolar disorder type I and type II using smartphones (bipolar disorder type I serve as reference), N = 84

	Model 1^a^			Model 2^a^			Model 3^a^		
	B	95% CI	p	B	95% CI	p	B	95% CI	p
A: Mood instability factor (MIF)	− 0.19	− 0.50; 0.025	0.51	− 0.25	− 0.81; 0.32	0.38	− 0.33	− 0.91; 0.26	0.27
B: Mood instability factor for depression (MIFD)	0.24	− 0.025; 0.50	0.076	0.25	0.011; 0.51	0.061	0.27	0.007; 0.53	*0.044*
C: Mood instability factor for mania (MIFM)	− 0.11	− 0.46; 0.24	0.53	− 0.15	− 0.50; 0.20	0.40	− 0.20	− 0.60; 0.20	0.32
D: Mood symptomatic factor for depression (MSFD)	0.30	− 0.37; 0.98	0.37	0.26	− 0.39; 0.92	0.43	0.26	− 0.39; 0.92	0.26
E: Mood symptomatic factor for mania (MSFM)	− 0.24	− 0.49; 0.025	0.076	− 0.24	− 0.52; 0.029	0.080	− 0.33	− 0.64; − 0.021	*0.037*
F: Mood intensity factor for depression (MIntFD)	0.36	− 0.72; 1.43	0.51	0.29	0.75; 1.34	0.58	0.29	0.75; 1.34	0.58
G: Mood intensity factor for mania (MIntFM)	− 0.24	0.50; 0.025	0.076	− 0.24	− 0.50; 0.023	0.073	− 0.29	− 0.56; − 0.10	*0.042*
H: Mood instability score (MIS)	0.037	− 0.53; 0.61	0.90	− 0.064	− 0.66; 0.53	0.83	− 0.064	− 0.66; 0.53	0.83

Italic values indicate statistical significance level (p value)

A: MIF = number of mood changes/number of weeks followed; B: MIFD = number of mood changes of depressive polarity/number of weeks followed; C: MIFM = number of mood changes of manic polarity/number of weeks followed; D: MSFD = number of days with depressive symptoms < − 0.5 on a mood scale from − 3 to + 3/number of weeks followed; E: MSFM = number of days with manic symptoms > 0.5 on a mood scale from − 3 to + 3/number of weeks followed; F: MIntFD: the summaric score of depressive symptoms/number of weeks followed; G: MintFM: the summaric score of manic symptoms/number of weeks followed; H: MIS: scores were computed for each user by applying the root mean square successive difference (rMSSD) method, taking the square root of the sum of the squared differences between daily and previous day mood scores

^a^Model 1: unadjusted. Model 2: adjusted for age and gender. Model 3: adjusted for age, gender, illness duration and psychopharmacological treatment (anticonvulsant treatment yes/no, antipsychotics treatment yes/no, antidepressant treatment yes/no, lithium treatment yes/no)

There was no significant correlation between mood instability factor for depression and self-monitored sleep duration (*B:* − *0.01, 95% CI* − *0.040; 0.02, p *= *0.44*). Furthermore, patients with bipolar disorder type II had statistically significantly lower Mood symptomatic factor for mania and mood intensity factor for mania (mood intensity factor for mania: models adjusted for age, gender, illness duration and psychopharmacological treatment (anticonvulsant treatment yes/no, antipsychotic treatment yes/no, antidepressant treatment yes/no, lithium treatment yes/no): *B:* − *0.074, 95% CI* − *0.15;* − *0.0022, p *= *0.043*) compared with patients with bipolar disorder type I. There were no differences in mood instability factor, mood instability factor for mania, mood symptomatic factor for depression, mood intensity factor depression and mood instability score between patients with bipolar disorder type I and bipolar disorder type II.

## Discussion

Apart from one previous study by the authors (Faurholt-Jepsen et al. 2015a), this is the first study investigating differences in daily smartphone-based self-monitored data collected over 9 months on illness activity in patients with bipolar disorder type I and bipolar disorder type II. As hypothesized we found that patients with bipolar disorder type II experienced higher mood instability for depression compared with patients with bipolar disorder type I, but lower intensity of manic symptoms. In addition, we found that in the entire sample regardless the bipolar disorder subtype, the patients had a mean mood level below zero during the 9-month study period and reported mixed symptoms a large proportion of time when experiencing levels of depression or mania. Regardless the bipolar subtype, the patients were able to validly evaluate their level of depressive and manic symptoms.

The finding that patients with bipolar disorder type II experienced more instability for depressive symptoms compared with patients with bipolar disorder type I is in line with findings from other studies including a study from our group suggesting that bipolar disorder type II is not simply a less serious subtype of bipolar disorder (Bopp et al. 2010; Judd et al. 2003; Joffe et al. 2004; Kupka et al. 2007; Vinberg et al. 2017). During the last decades there has been an increased shift from a focus on awareness of the impact of inter-episodic mood instability (MacQueen et al. 2003; Harrison et al. 2016), and a substantial proportion of patients with bipolar disorder experience subsyndromal mood swings on a daily basis even though they overall seem to be in remission. Mood instability is associated with poor prognostic factors including impaired functioning, increased risk of hospitalization, high risk of relapse, and comorbid substance use disorder (Bopp et al. 2010; Judd et al. 2003; Joffe et al. 2004; Kupka et al. 2007; Strejilevich et al. 2013; Patel et al. 2015; O’Donnell et al. 2018; Gershon and Eidelman 2015). The patients in the present study had a mean illness duration of 18.0 years (SD 10.1) and several prior depressive and manic episodes. Studies investigating whether differences in mood instability between bipolar disorder type I and bipolar disorder type II are intrinsic to bipolar disorder or a consequence of illness progression have not been conducted. However, illness duration was included as a possible confounder in the statistical analyses in the present study and did not alter the estimates. Smartphone-based monitoring of mood and mood instability in high-risk groups such as the adolescent and emerging adult offspring of parents with bipolar disorder may be a fruitful new direction of early identification and characterization of the prospective course of mood instability (Duffy et al. 2018; Kessing et al. 2017).

Mood instability may predict worse long-term clinical course and functioning beyond what is predicted by prior and current depressive and/or (hypo)manic episodes (Strejilevich et al. 2013; McKnight et al. 2017) supporting the idea that different measures of subsyndromal illness activity may be a more sensitive outcome measure in maintenance RCTs than for example time to first relapse or recurrence of affective episodes (Bopp et al. 2010; Bonsall et al. 2012; Saunders et al. 2016; Broome et al. 2015). Thus, there is a need to identify and monitor inter-episodic symptoms giving the opportunity to intervene. Innovative RCT’s investigating the effect of treatment interventions on mood instability are ongoing and will hopefully provide more insight to this area (Saunders et al. 2016).

Throughout the present study period all patients had continuous contact to a study nurse through a bi-directional feedback loop based on changes in the smartphone-based self-monitored data incorporated in the MONARCA II system (Faurholt-Jepsen et al. 2014). However, despite this rather intensive intervention they presented with subsyndromal levels of symptoms during large proportions of time, reflecting the serious and chronic nature of bipolar disorder.

In contrast to our hypotheses and in contrast to findings from our previous study patients with bipolar disorder type II had a lower mean mood level or experienced higher levels and intensity of depressive symptoms compared with patient with bipolar disorder type I (Faurholt-Jepsen et al. 2015a). The findings on mood instability for depression and mood symptomatic factor for mania from the present study are in line with findings from our previous study. However, in contrast to our previous study, the present patients with bipolar disorder type II did not experience higher mood symptomatic factor for depression and mood intensity factor for depression factor. Differences in findings between the two studies may be due to differences in the included samples in the two studies. Patients in our previous study were recruited early during their course of treatment, whereas patients in the present report had received two full years of outpatient treatment comprising combined evidence-based psychopharmacological treatment and supporting therapy, including psychoeducation at the specialized outpatient clinic. In our previous study a total of 27.2% of the patients were prescribed with antidepressants and 57.6% were prescribed with lithium, whereas in the present study a total of 22.6% were prescribed with antidepressants and 63.1% were prescribed with lithium. In addition, a larger proportion of the patients in the present study were in full time employment compared with the patients from the previous study (17.7% vs. 8.6%).

### Limitations

Some limitations to the present report should be mentioned. First, the patients included in the study were recruited as part of a larger RCT and thus not recruited according the bipolar disorder subtype. The unequal group size between the two disorders was therefore not intended. The statistical power for the study was calculated for the original RCT and consequently power calculations were not conducted for the present report (Faurholt-Jepsen et al. 2015a). We did not correct for multiple testing since the study is considered as hypotheses generating and the results needs further replication. However, it should be noted that if Bonferroni corrections were applied none of the models would be statistically significant. Second, regarding the generalizability of the results, the patients included in the present study had previously been treated at The Copenhagen Clinic for Affective Disorders, Denmark which is a specialized outpatient clinic in the period 2004 to January 2016. Along this line, it cannot be excluded that the patients who agree to participate in the present study may represent a subgroup of patients with bipolar disorder. Third, the patients presented with rather low levels of depressive and manic symptoms, and received different types, doses and combinations of psychopharmacological treatment during the study. Since a higher proportion of patients with bipolar disorder type I received antipsychotics compared with patients with bipolar disorder type II, the statistical analyses included the different psychopharmacological treatments prescribed (anticonvulsant treatment yes/no, antipsychotic treatment yes/no, antidepressant treatment yes/no, lithium treatment yes/no) as covariates. However, residual confounding due to differences in psychopharmacological treatment between the two groups cannot be excluded. It is likely that differences in psychopharmacological treatment (antipsychotics) may affect differences in mood instability and should be investigated further in larger studies. Fourth, to date there are no clear consensus on the definition, measurement, use and reporting of mood instability in patients with bipolar disorder, and thus the measures used in the present report were a combination of measures used by others and measures used by the authors in a previous study (Strejilevich et al. 2013; Faurholt-Jepsen et al. 2015a; O’Donnell et al. 2018; Ebner-Priemer and Trull 2009). Using, defining, measuring and reporting mood instability in a standardized way across studies could improve the quality, reproducibility and comparison of results between studies. Fifth, we did not use last-observation-carried-forward method or imputation techniques to handle missing data since there was a high adherence to self-monitoring during the study. The patients presented with rather low level of depressive and manic symptoms and therefore data is estimated to be missing at random. It cannot be excluded that patients did not conduct the self-monitoring during the most severe time point. However, the patients were able to validly evaluated their level of depressive and manic symptoms. Investigating the validity of self-monitored symptoms during more severe affective episodes could be a target for future studies.

Sixth, it may be that systematic self-monitoring and reporting of mood could potentially in itself affect mood and mood awareness. Along this line, the risk of patients using the smartphone-based self-monitoring system systematically reported lower mood, to gain attention from the study nurse leading to non-random measurement error, must be considered. However, our previous studies using the same smartphone-based self-monitoring system have showed neither positive nor negative effects of smartphone-based self-monitoring, and that the self-monitored mood levels correlated statistically significant with clinically rated depressive and manic symptoms measured using the Hamilton Depression Rating Scale and the Young Mania Rating Scale (Faurholt-Jepsen et al. 2015b, 2015c).

### Perspectives

Smartphones are ubiquitous and reflect an unobtrusive way of collecting fine-grained real-time data during naturalistic settings on the course of illness and detailed characterization of prodromal and subsyndromal symptoms of depression and (hypo)mania in patients with bipolar disorder. Measures of mood instability may be a promising measure of inter-episodic illness activity in bipolar disorder and may be a more sensitive outcome measure in maintenance RCTs than for example time to first relapse or recurrence of affective episodes since patients continue to experience subsyndromal mood swings.

### Conclusions

Patients with bipolar disorder type II experienced higher mood instability for depression compared with patients with bipolar disorder type I. Patients with bipolar disorder type I and II experienced mixed symptoms a large proportion of time. These findings emphasize the need to monitor, identify and treat subsyndromal inter-episodic symptoms. Future studies investigating the effect of treatment on mood instability measures are needed.
